# M1 Corticospinal Mirror Neurons and Their Role in Movement Suppression during Action Observation

**DOI:** 10.1016/j.cub.2012.12.006

**Published:** 2013-02-04

**Authors:** Ganesh Vigneswaran, Roland Philipp, Roger N. Lemon, Alexander Kraskov

**Affiliations:** 1 Sobell Department of Motor Neuroscience and Movement Disorders, UCL Institute of Neurology, Queen Square, London WC1N 3BG, UK

## Abstract

Evidence is accumulating that neurons in primary motor cortex (M1) respond during action observation [1, 2], a property first shown for mirror neurons in monkey premotor cortex [3]. We now show for the first time that the discharge of a major class of M1 output neuron, the pyramidal tract neuron (PTN), is modulated during observation of precision grip by a human experimenter. We recorded 132 PTNs in the hand area of two adult macaques, of which 65 (49%) showed mirror-like activity. Many (38 of 65) increased their discharge during observation (facilitation-type mirror neuron), but a substantial number (27 of 65) exhibited reduced discharge or stopped firing (suppression-type). Simultaneous recordings from arm, hand, and digit muscles confirmed the complete absence of detectable muscle activity during observation. We compared the discharge of the same population of neurons during active grasp by the monkeys. We found that facilitation neurons were only half as active for action observation as for action execution, and that suppression neurons reversed their activity pattern and were actually facilitated during execution. Thus, although many M1 output neurons are active during action observation, M1 direct input to spinal circuitry is either reduced or abolished and may not be sufficient to produce overt muscle activity.

## Highlights

► PTNs in primary motor cortex show mirror properties ► These neurons project to the spinal cord, but no overt muscle activity was detected ► Compared with execution, mirror PTNs reduce or abolish discharge during observation ► Disfacilitation of mirror PTNs during observation may prevent unintended movement

## Results and Discussion

Mirror neurons are particularly fascinating in that they are activated not only by one’s own actions but also by the actions of others. Mirror neurons in macaque area F5 were originally shown to respond during both the monkey’s own grasping action and during observation of grasp carried out by a human experimenter [3, 4]. Recordings made in adjacent primary motor cortex (M1) were reported as lacking mirror-like activity, and this was taken as evidence that the monkey was not making covert movements while it observed actions. This conclusion was very much based on the idea that M1, unlike premotor cortex, is an “executive” structure, whose activity has many “muscle-like” features, which can be reliably linked to the production of movement [5–8].

However, since 1996, evidence has been steadily accumulating for the presence of mirror-like activity in M1, in both monkeys [1, 2] and humans [9–13]. This activity has been open to a number of interpretations, including a role for M1 as part of a frontal network involved in mental rehearsal or simulation of the observed action [14].

The executive role of M1 in the brain’s motor network is strongly supported by the architecture of its outputs to the spinal cord [7, 15–17], so it is a challenge to explain why the presence of extensive mirror-like activity within M1 does not lead to movement. To understand this, we recorded from identified corticospinal neurons in M1 and showed that, although many of these neurons exhibit mirror-like activity, there were major differences in their pattern and extent of discharge during action execution versus action observation.

We trained two adult, purpose-bred macaque monkeys to either perform a precision grip between index finger and thumb or watch an experimenter perform the same grip. In one monkey (M43), in which the initial findings were made, the design was very simple: the monkey either grasped a small piece of fruit placed within its peripersonal space or watched the experimenter, sitting opposite the monkey, grasp a similar reward placed in the monkey’s extrapersonal space.

**Figure 1:**
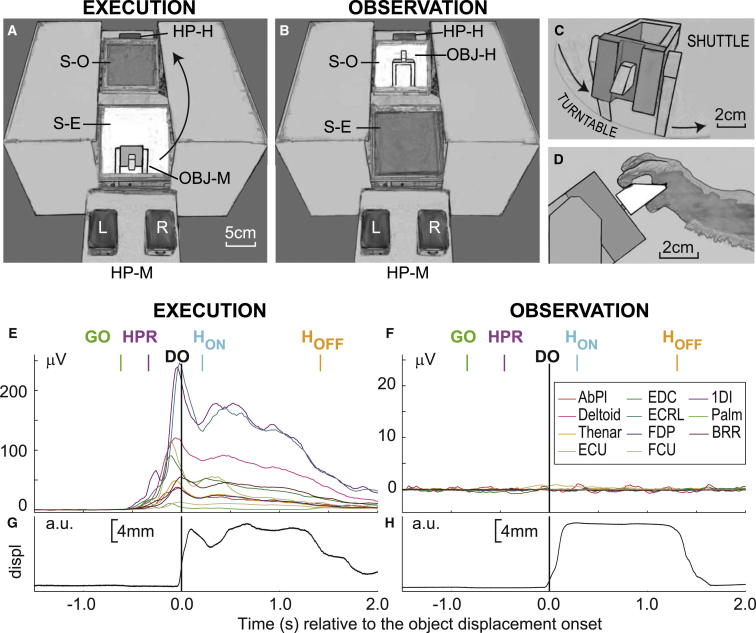
Experimental Apparatus (A and B) The diagram shows the monkey’s perspective of a carousel device used to present an object during execution (A) or observation trials (B). Notations: HP-M, homepads monkey, left (L) and right (R); HP-H, homepad experimenter; S-E and S-O, screens that could be electronically switched from opaque to transparent during execution (S-E) or observation trials (S-O), allowing the monkey a direct view of the object (OBJ-M) when the monkey grasped it (A) and of the same object (OBJ-H) when the experimenter grasped it (B). (C) Close-up of the trapezoid object (affords precision grip) mounted on a spring-loaded shuttle. (D) Side view of monkey grasping the trapezoid object using precision grip. (E–H) Average EMG traces from 11 hand or arm muscles from one session in monkey M47 for execution (E) and observation trials (F). During execution, all muscles were active, but there was no modulation during observation. Note that a ten times higher gain was used for observation trials to emphasize absence of EMG activity (note different y scale). Averages are aligned to the onset of the object displacement (DO) by the monkey (E) or human (F). Average displacement of the object is shown for execution and observation trials in (G) and (H), respectively. The median times of other recorded events relative to DO are shown as vertical lines above. Muscles are color-coded as shown in key at right of (F) and are abbreviated as follows: AbPl, abductor pollicis longus; deltoid; thenar; ECU, extensor carpi ulnaris; EDC, extensor digitorum communis; ECRL, extensor carpi radialis longus; FDP, flexor digitorum profundus; FCU, flexor carpi ulnaris; 1DI, first dorsal interosseous; Palm, palmaris; BRR, brachioradialis. Notations: GO, go cue; HPR, homepad release; H_ON_, stable hold onset; H_OFF_, stable hold offset.

In the second monkey (M47), we wanted to compare M1 mirror neuron activity for grasp of exactly the same object, whether made by monkey or experimenter. We designed a device that required the monkey to grasp a small trapezoidal object placed in front of it (OBJ-M; Figure 1A) with a precision grip (Figure 1D), then displace it by a controlled amount and hold it steady for 1 s (Figure 1G). The monkey then released the object and received a reward. We compared activity during these execution trials with interleaved observation trials. In the latter, the same object was presented to the experimenter, who sat opposite the monkey (Figure 1B). The experimenter gripped, displaced, and held the object (Figure 1H) in the same way as the monkey.

Each trial began with both monkey and experimenter gently depressing homepad switches (Figures 1A and 1B; HP-M [monkey] and HP-H [human], respectively). After a short delay, an electronically operated screen turned from opaque to transparent; for execution trials, this was the screen nearest to the monkey (screen execution [S-E]; Figure 1A), allowing the monkey to see the object in front of it. After a variable delay (0.8–1.5 s), the illumination around the object changed to a green color, and this acted as a GO signal (Figure 1E), cueing the monkey to release the right homepad (homepad release [HPR]-R) and reach, grasp, and displace the object (displacement onset [DO]; Figures 1E and 1G). After the monkey had held the object for ∼1 s (H_ON_ to H_OFF_), it released it and received a reward. For observation trials, the screen furthest from the monkey was cleared (screen observation [S-O]; Figure 1B), allowing the monkey to see the entire action carried out by the experimenter. Again, an LED GO signal cued the experimenter to release her homepad (Figure 1F, HPR) and then reach, grasp, displace, and hold the object (Figure 1H). Monkeys were rewarded after both execution and observation trials. In addition, monkey M47 was trained to grasp a sphere (whole-hand grip) and a ring (hook grip). The results of detailed comparison between these three grips will be the subject of a future publication, but we note here that we found grasp-related differences in the activity of many mirror pyramidal tract neurons (PTNs) during both grasp execution and grasp observation.

A total of 132 PTNs were recorded from M1 in the two monkeys (M43, 79 PTNs; M47, 53 PTNs). In both monkeys, we recorded from up to 11 different arm, hand, or digit muscles to confirm that the monkey did not make covert movements as it watched the experimenter [18]. Electromyogram (EMG) recordings during execution all showed activity but were silent during observation (Figure 1F; note difference in gain).

**Figure 2:**
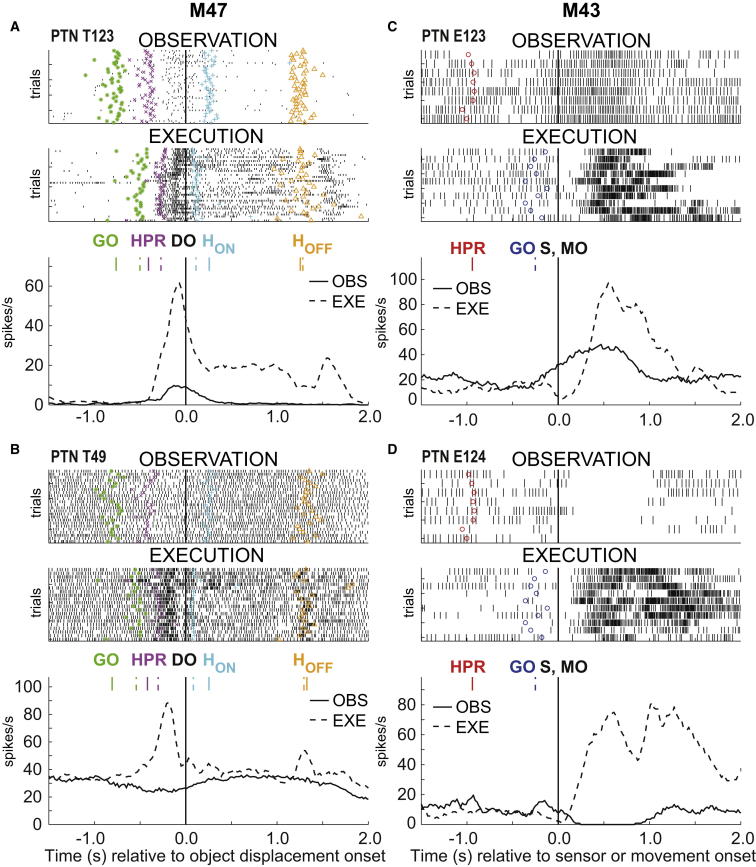
Mirror PTNs in M1 Examples of M1 facilitation (A and C) and suppression (B and D) mirror PTNs in M47 (A and B) and M43 (C and D). Each panel consists of raster plots for observation and execution trials and corresponding histograms (solid and dashed lines, respectively). Histograms were compiled in 20 ms bins and then smoothed using a 140 ms sliding window. In (A) and (B), all data were aligned to onset of the object displacement (DO); other behavioral events are indicated by colored markers for each trial on raster plots and with vertical lines on histograms (cf. Figure 1). In (C) and (D), all execution trial data were aligned to movement onset (MO), defined using onset of biceps EMG activity. All observation trial data were aligned to a sensor signal (S), which detected first contact of the experimenter with the object. HPR indicates beginning of the experimenter’s movement in observation trials. GO markers indicate the cue for the monkey to grasp the reward in execution trials.

In total, 77 of 132 PTNs (58%) showed significant modulation during action observation. Figure 2 shows examples of mirror neurons. These were classified either as “facilitation”-type mirror neurons, which increased discharge during observation trials (cf. [3]; Figures 2A and 2C), or as “suppression”-type, in which discharge was reduced or abolished during observation (cf. [18]; Figures 2B and 2D). The key events in each trial are shown by colored symbols superimposed on the rasters of unit activity. For M47 (Figures 2A and 2B), the rasters are aligned to object displacement onset for both conditions (cf. Figure 1). The facilitation PTN shown in Figure 2A became active soon after HPR, but the activation was much more pronounced for execution (dashed line in averaged spike activity) than for observation (solid lines).

The suppression mirror neuron shown in Figure 2B had a steady baseline discharge of around 30–35 spikes/s, which decreased soon after the GO signal in the observation condition. In striking contrast, it showed a marked increase in discharge during execution up to a peak of 90 spikes/s: it reversed its pattern of activity as the task changed from observation to execution.

In M43, the task was more naturalistic and the task events somewhat less well defined (see Experimental Procedures). Nevertheless, for observation trials, a contact sensor signal allowed us to align rasters with the moment the experimenter first grasped the piece of fruit. The facilitation PTN shown in Figure 2C increased its discharge shortly before the experimenter’s grasp and peaked around 500 ms after it. For execution trials, rasters were aligned with the onset of the monkey’s muscle activity (see Experimental Procedures); this PTN showed a complex pattern of early suppression followed by later activation, which was again much greater than the peak rate during observation (95 versus 45 spikes/s). The PTN shown in Figure 2D had a baseline firing rate of around 10 spikes/s, which was completely suppressed during observation, whereas it showed pronounced activity (peak of 75 spikes/s) late in the monkey’s own reach-to-grasp.

**Figure 3:**
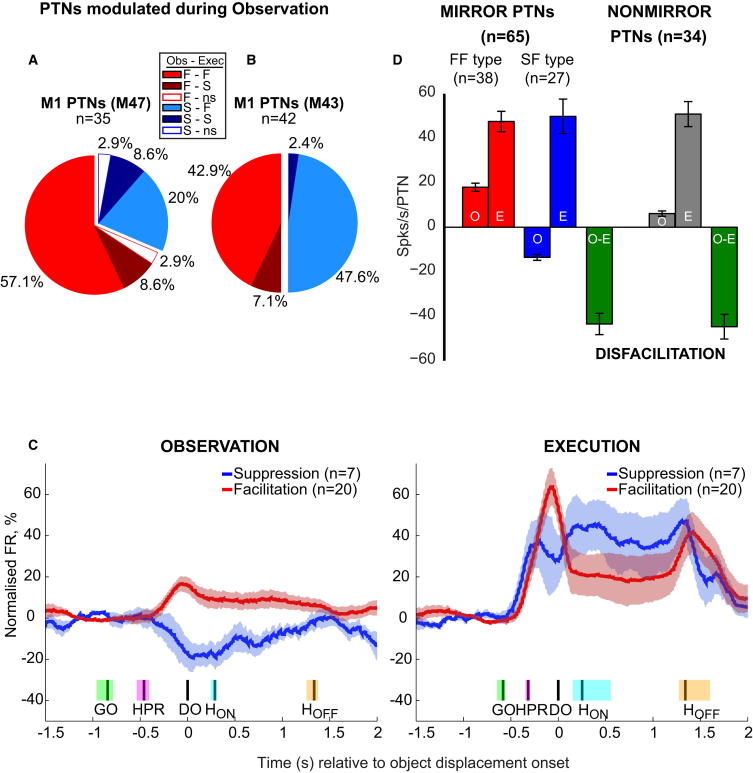
Population Activity of M1 Mirror Neurons (A and B) Pie charts showing different types of facilitation (red, F) and suppression (blue, S) PTNs recorded during action observation (Obs in inset box) in M47 (A) and M43 (B). Lighter shades of both colors indicate proportions of these neurons whose discharge was facilitated during execution (Exec in inset box); darker shades indicate proportions showing suppression during execution (a relatively small proportion). ns, nonsignificant change in modulation during execution. (C) Left: population averages during observation for corticospinal mirror neurons (M47) that were activated during execution and whose discharge was significantly suppressed (blue) or facilitated (red) during observation (together with SEM, shaded areas). Firing rates were normalized to the absolute maximum of the smoothed averaged firing rate of individual neurons defined during execution and observation trials, and baseline firing rate was subtracted. Data aligned to DO, the median (black line), and the 25^th^ to 75^th^ percentile times of other events recorded are shown as shaded areas: GO (green), HPR (magenta), hold H_ON_ (cyan), and H_OFF_ (yellow). Firing rates were smoothed using a 400 ms sliding window in 20 ms steps. Right: population average for the same groups of mirror neurons during execution. Facilitation-type PTNs showed higher discharge rates during execution compared with observation trials, and suppression type PTNs changed pattern to facilitation during execution. (D) Maximum firing rate of PTNs during observation and execution trials, expressed as raw firing rates (with SEM). Results from both monkeys were pooled. Red bars show average rates for 38 M1 PTNs facilitated during both observation (O) and execution (E) (F-F type). Note the much lower rate during observation. Blue bars show rates for 27 M1 PTNs suppressed during observation (O) and facilitated during execution (E) (S-F type). The left green bar shows the mean firing rate for all these mirror PTNs in observation minus that in execution, to capture the total amount of disfacilitation in the output from these neurons that occurred during observation. On the right are similar results for PTNs that did not show any mirror activity.

Figure 3 shows the population analysis of M1 PTNs modulated during observation (n = 77). In M47, we recorded 35 PTNs (Figure 3A), of which the majority (24 of 35, 68.6%) were facilitated during observation (Obs, F), and most of these (20 of 35, 57.1%) were also facilitated during execution (Exec, F-F type, red). A few PTNs either showed suppression (F-S, three PTNs, 8.6%, dark red) or were nonsignificant (ns; one PTN, 2.9%) during execution. The remaining 11 of 35 PTNs (31.4%) showed suppression during observation; 7 of 35 (20%) were facilitated during execution (S-F, light blue), with a few also suppressed (S-S, three PTNs, 8.6%) or nonsignificant (one PTN, 2.9%) during execution.

Similar results were found in M43 (Figure 3B): again, many PTNs (21 of 42, 50%) showed facilitation during observation, and most were also facilitated during execution (18 of 42, 42.9%). Almost all PTNs exhibiting suppression during observation (21 of 42, 50%) reversed their activity and were facilitated during execution (20 of 42, 47.6%). Note that of the 77 PTNs shown in Figures 3A and 3B, only 65 would be strictly classified as mirror neurons, i.e., PTNs which were either facilitated or suppressed during observation and facilitated during execution.

Figure 3C compares the time-resolved normalized firing rates of mirror neurons during observation and execution (M47). We selected the two main subgroups of PTNs: facilitation mirror neurons that were also facilitated during execution (n = 20 F-F type PTNs, red traces in Figure 3C) and suppression mirror neurons, which reversed their firing pattern and were also facilitated during execution (n = 7 S-F PTNs, blue traces). During observation (shown at left), both subgroups modulated their background firing rate shortly after the experimenter’s HPR, with peak modulation at DO. During execution (shown at right), facilitation PTNs were around three times as active compared with observation; discharge increased to 64% of the maximum modulation above baseline (see Experimental Procedures) versus only 17% during observation. The subgroup of suppression PTNs reversed their pattern of discharge from 19% of the maximum modulation below baseline for observation to 47% above it for execution. Changes in firing rate were sustained at lower levels during the hold period.

Similar patterns were found in M43. For facilitation mirror neurons (F-F type, n = 18), discharge during execution was 60% of the maximum modulation above baseline versus 44% for observation. Suppression mirror neurons (S-F type, n = 20) discharged at 31% below baseline during observation but reversed to 63% above it for execution.

In Figure 3D, we estimate changes in maximum firing rates (nonnormalized) when the task switched from observation to execution. Pooling data from both monkeys, we calculated the mean firing rate for 38 F-F type mirror neurons (red bars), i.e., those facilitated during execution (E) and strongly attenuated during observation (O). The blue bars represent 27 S-F type PTNs, which were suppressed for observation but facilitated for execution. The difference in mean firing rate of facilitation versus suppression PTNs in observation was around 5 spikes/s/PTN. The first green bar combines results from these two sets of mirror neurons and shows that, compared with the execution condition, the population mean firing rate during observation represented a mean disfacilitation of around 45 spikes/s/PTN. On the right of Figure 3D, we estimated the same change for a group of 34 nonmirror PTNs recorded in the same monkeys. By definition, these PTNs showed no significant modulation during observation, so they were also effectively disfacilitated during observation.

Finally, in M47, we carried out spike-triggered averaging to determine whether PTNs, whose discharge was modulated during action observation, also exerted postspike facilitation of hand muscles, identifying them as corticomotoneuronal cells [16, 19]. Of the 34 mirror PTNs tested, five (15%) had clear postspike effects; three were facilitation and two were suppression mirror neurons.

This study reveals that, during observation of precision grip, there is modest but widespread mirror-like activity among corticospinal neurons in the hand area of macaque primary motor cortex. Significant modulation of discharge during action observation was seen in over half of the recorded PTNs. Most of these (38 of 65, 58.5%) were categorized as “facilitation” mirror neurons, similar to those originally described by Gallese et al. [3], increasing their discharge during both observation and execution. However, these neurons were far less active for observation than execution (Figures 3C and 3D), with the overall normalized firing rate down to less than half of that when the monkey performed the grip. This comparison is valid in that both human and monkey performed a similar set of actions on the same trapezoid object, and both used a precision grip. There were some differences in the kinematics, with the monkey moving more rapidly than the human (Figure 1G versus Figure 1H); however, this is unlikely to explain the difference in firing rate, because we could not find any consistent correlation between firing rate and movement time across execution and observation trials (see Supplemental Experimental Procedures available online).

Just as we had previously demonstrated in area F5 of the premotor cortex [18], we also found a significant proportion of “suppression” mirror neurons in M1 (27 of 65, 41.5%). During action observation, these neurons either decreased their firing rate (solid line in Figure 2B) or stopped firing altogether (Figure 2D). Nearly all of these “suppression” PTNs reversed their pattern of activity during execution and increased their firing rate. It is worth noting that this change in pattern could not be explained by any differences in the kinematics of human versus monkey action.

It is of course of interest to compare mirror neuron activity in F5 and M1. A preliminary comparison of PTN activity in M43, in which many M1 and F5 recordings were obtained simultaneously, suggests important differences between F5 and M1. In F5, facilitation mirror neuron activity was similar during observation and execution, whereas in M1, PTN activity was relatively attenuated during observation. A previous study [2] showed that activity in unidentified M1 neurons evoked by observation of a human experimenter performing a reaching task was much lower than in execution trials. Several human fMRI studies have reported much lower levels of activation for action observation than execution [13, 20]. The lower level of activity in M1 during action observation might explain why some fMRI studies have not reported significant activation in this area (monkey [21, 22]; human [20]).

Although our knowledge of cortical changes during observation is quite advanced, our knowledge of the effect of these changes on the spinal circuits for movement is still rudimentary [11]. The impact on the spinal motor system of mirror-neuron PTN activity in F5 versus M1 outputs is likely to be fundamentally different. M1 contributes 50% of the descending corticospinal projection from the frontal lobe [15], terminates heavily in the lower cervical cord [19], and includes direct corticomotoneuronal projections influencing digit muscles [7]. In contrast, area F5 contributes only 4% to the frontal lobe corticospinal projection and terminates mainly in the upper cervical cord [23, 24]. The executive or “muscle-like” status of neuronal activity in M1 has been repeatedly emphasized [5–8].

During observation, discharge in M1 facilitation mirror PTNs was attenuated and even reversed in suppression mirror PTNs. Taken together, these findings would mean that M1 output to spinal interneurons and motoneurons involved in generating movements in hand and digit muscles could be strongly disfacilitated during observation (green bars in Figure 3D). Metabolic activity in monkey spinal cord has been reported to be depressed during action observation [25]; although this could reflect active inhibition, it could presumably also have resulted from a disfacilitation of descending excitation as described here.

We do not know whether the effects at the spinal level of our sample of mirror PTNs were excitatory, inhibitory, or mixed [16, 26]. There is one notable exception to this, namely the mirror PTNs identified as corticomotoneuronal cells [7]. Because the synaptic terminals of these cells on spinal motoneurons are not subject to presynaptic inhibition [27], there is no obvious mechanism to prevent discharge in these cells from facilitating their target motoneurons. So it is interesting that two of the five corticomotoneuronal cells that we identified showed suppression of activity during observation. Such a mechanism might help to prevent this input from contributing to unwanted discharge of motoneurons and movement. Suppression of discharge was also seen for a small population of PTNs during execution trials (dark colors in Figures 3A and 3B); PTN disfacilitation has been reported before for tasks requiring skilled movements of the digits [19], including tool use [28].

Why are M1 output neurons modulated during action observation? If M1 is considered to be part of a larger “action observation network” [10, 29], then it is not surprising that the output neurons, which are strongly embedded in the intrinsic cortical circuitry [30, 31], are also modulated. However, because of the functional proximity of M1 corticospinal neurons to the spinal apparatus, to avoid overflow of their activity into unintended, overt movements during processes that involve action observation, it may be important to attenuate or block that activity. This may involve inhibitory systems operating at both cortical [32, 33] and subcortical levels [34]. Viewed in this way, action observation is yet another manifestation of the dissociability of motor cortex and muscle activity ([35–38]; recently reviewed in [39]).

These findings show for the first time that PTNs in primary motor cortex exhibit mirror activity when monkeys watch humans grasping. The presence of this activity in the corticospinal output must have consequences for spinal networks supporting voluntary movements. The striking differences between M1 PTN activity for observation versus execution may help us understand more about the patterns of PTN discharge that lead to movement, as well as those that do not. They may also help to explain why we do not imitate every action that we observe.

## Experimental Procedures

All experimental procedures were approved by the local ethical procedures committee and carried out in accordance with the UK Animals (Scientific Procedures) Act. Experiments involved two adult purpose-bred *Macaca mulatta* monkeys (M43, female, 5.5 kg; M47, male, 5.0 kg).

### Task

In a simple version of the experiment, a precision grip was used by either the experimenter or monkey (M43) to grasp a small food reward placed on a table between them (see [18]). Trials for action execution began when the experimenter released a homepad (HPR) on her side of the table and placed a small food reward on the table, close to the monkey’s hand. The experimenter’s HPR cued the monkey’s reach-to-grasp. For action observation, a small piece of food was placed on the table beyond the monkey’s reach. The experimenter released her homepad (HPR), approached the food, and grasped and held it in a precision grip. The experimenter wore a glove containing a magnet at the tip of the index finger. As the experimenter approached the reward, a magnetic sensor in the table generated a sensor pulse. Trials were repeated once every 4–5 s in a block of 10, and on average, the monkey was rewarded after every fifth trial.

For the experiment involving M47, the monkey sat facing a human experimenter with the carousel device between them (Figure 1). Each execution trial (Figure 1E) began with the monkey resting both hands on their respective homepads (Figure 1A, HP-M: monkey). After a short delay (∼0.8 s), a trapezoid-shaped object (Figure 1C) on the monkey’s side of the carousel (Figure 1A, OBJ-M) became visible when an opaque screen (Figure 1A, S-E), placed in the monkey’s line of sight with the object, was electronically switched to become transparent. After a variable time period (0.8–1.5 s), a green LED came on, changing the illumination around the object and acting as a GO signal for the monkey to release its right hand from the homepad (Figure 1E, GO, HPR), reach out and grasp the trapezoid in a precision grip, and displace it (Figure 1D; Supplemental Experimental Procedures). A signal was generated at DO (Figure 1E). The monkey held the object steadily for 1 s and then released it (H_ON_ to H_OFF_), replaced his hand on the right homepad, and received a reward.

During observation trials, which were interleaved with execution trials using a pseudorandom process, the roles were simply reversed. The carousel turned so that the same object was now on the experimenter’s side. The trial began when all homepads were depressed. After a small delay, the object became visible to the monkey, who viewed it through a second switched screen (Figure 1B, S-O). The green LED now cued to the experimenter to GO, release their homepad (Figure 1B, HP-H), reach and grasp the object, displace it and hold it for 1 s, and then release it (see Figure 1H). The monkey was also rewarded at the end of each observation trial.

The carousel device allowed us to determine the precise timing of each event making up the whole action. Although the human and monkey grasps were very similar, the monkey’s movements were faster than the experimenter’s: from HPR to DO was typically 0.31 s for the monkey and 0.45 s for the human.

Each neuron was also recorded during execution and observation of two other grasps, involving different objects mounted on the carousel: a sphere and a ring. We are only presenting data on the precision grip in this report.

### Electrophysiological Recordings and Stimulation

When fully trained, monkeys were prepared for single neuron cortical recordings using a Thomas Recording 16-channel drive ([18]; Supplemental Experimental Procedures). PTNs were identified by searching for antidromic responses to stimuli applied to chronically implanted pyramidal tract electrodes [18]. Neurons with invariant response latency and satisfying a collision test were identified as PTNs. PTNs were not tested for their task-related activity until antidromic identification had been completed, so the sample is unbiased in terms of activity.

PTNs were recorded in 27 and 40 sessions in M43 and M47, respectively, and over a period of 25 and 10 weeks, respectively. PTNs were recorded for a minimum of ten observation and ten execution trials. Most recordings were from large, fast PTNs: antidromic latencies ranged from 0.51 to 5.35 ms (median 1.05 ms) [40]. Most PTNs were recorded from tracks in the M1 hand region close to the central sulcus and at sites from which digit movements could be evoked with low-threshold intracortical microstimulation (<20 μA, 79%; <10 μA, 55%).

We recorded EMGs from 9 (M43) or 11 (M47) arm, hand, and digit muscles using chronically implanted electrodes (Figure 1). Eye movements were recorded with a noninvasive ISCAN camera system (ETL-200, 120 Hz). We found no correlation between the pattern of PTN activity and eye movements (Supplemental Experimental Procedures).

### Analysis of PTN Activity

PTNs were discriminated and clustered using modified Wave_clus software [41] and carefully tested to ensure that only spikes from the identified PTN were used in analysis; statistical tests were then carried out to detect modulation of PTN discharge from baseline in different periods of the execution and observation tasks (Supplemental Experimental Procedures). Only PTNs that showed significant (p < 0.05) modulation in the firing rate during observation were retained to construct the population averages of PTNs with “mirror-like” activity (Figure 3). Population averages were expressed as a percentage of a PTN’s maximum modulation above or below baseline. Corticomotoneuronal cells were identified by spike-triggered averaging of EMG (Supplemental Experimental Procedures).

## Supplemental Information

Document S1. Supplemental Experimental Procedures
