# Supplementary material for: M1 Corticospinal Mirror Neurons and Their Role in Movement Suppression during Action Observation
Source: Curr Biol. 2013 Feb 4;23(3):236–43. doi: 10.1016/j.cub.2012.12.006 (PMC3566480; doi:10.1016/j.cub.2012.12.006)
Supplement: Document S1. Supplemental Experimental Procedures [file mmc1.pdf]

Current Biology, Volume *23*

Supplemental Information

M1 Corticospinal Mirror Neurons and Their Role in Movement Suppression during Action Observation

Ganesh Vigneswaran, Roland Philipp, Roger N. Lemon, and Alexander Kraskov

Supplemental Experimental Procedures

Task

M43

In this monkey, execution trials were carried out in blocks of ten trials, alternated with blocks of trials for action observation (see [1] for details). During execution trials, two timing events were available: firstly, the experimenter's release of her homepad (HPR), which was positioned on her side of the table. Each trial began with the experimenter's right hand resting on the homepad. About 1.5 s later a tone sounded which cued the experimenter to release the homepad and place a reward near the monkey's hand. The second signal was EMG activity recorded from the monkey's left arm and hand. The clearest burst of activity at movement onset was found in the biceps EMG, as the monkey lifted its arm off the table and reached to collect the food reward, so this signal (muscle onset MO, in Fig. 2C and D) was used to align the recorded data. During observation trials, we again recorded the experimenter's homepad release (HPR) and in addition a signal (S, Fig. 2C and D) from a magnetic sensor embedded in the table just below the point where the experimenter placed a food reward before each observation trial began. This point was in the monkey's extrapersonal space, around 42 cm from the monkey's edge of the table. The sensor was activated when a small magnet concealed in the finger tip of the experimenter's glove approached the reward. In this part of the study the experimenter grasped and held the reward but did not displace it.

M47

The carousel device consisted of a round turntable mounted on the shaft of a strong and silent DC motor. The trapezoid object (9 mm x 11 mm x 26 mm) was designed to elicit a precision grip between the tips of thumb and index finger and it was mounted on a shuttle fixed to the turntable. The object moved on a low-friction, spring-loaded shaft. Rotation of the turntable under computer control could present the object either to the monkey (execution trials) or experimenter (observation trials). For execution trials, the object was well within the monkey's peripersonal space and it could see the object when a screen (10.5 cm x 10.5 cm. S-E in Fig. 1A) was switched electronically from opaque to transparent.

Each execution trial began with the monkey resting both hands on their respective homepads. After a short delay (~0.8 s), the screen was made transparent. When an LED was activated (0.8-1.5 s later) it changed the background illumination of the object to green, signalling the monkey to GO. It

released its right hand (HPR) and reached out and grasped the object in a precision grip (Fig. 1C). The monkey was required to displace it by around 5-8 mm (Fig. 1G) into an electronically defined target zone, applying a load force of around 0.6 N and pulling the object upwards, towards the monkey. The correct extent of displacement was monitored by a Hall effect sensor on the shaft of the shuttle, and fed back as an audible tone to the monkey. The displacement onset (DO, Fig. 1E) was also determined from the Hall effect signal.

Around 1 s after the trial was completed, the monkey received a small piece of fruit as a reward at the end of each execution trial; this was delivered directly to the monkey's mouth. The monkey was also rewarded 1s after each observation trial.

Surgical Preparation

Three different surgical procedures were carried out under deep general anaesthesia and full aseptic precautions. In the first, a custom-fitted Tekapeek headpiece was surgically implanted to allow head restraint. In the second, carried out 12-15 weeks later, chronic electromyogram (EMG) patch electrodes were implanted in up to 11 arm, hand and digit muscles [2]. In the third surgery, 4-9 weeks later, a recording chamber was mounted over M1 and F5, and stimulating electrodes were chronically implanted in the medullary pyramid for subsequent antidromic identification of pyramidal tract neurons [1, 3].

Parameters for Recording and Stimulation

Single neurons: These were recorded with a 16 channel Thomas Recording drive, using glass-insulated platinum electrodes with tip impedances of 1-2 MOhm. Following pre-amplification (x20, Thomas Recording headstage amplifier), the signals from each electrode were further amplified (typically x150) and broadly band-pass filtered (1.5 Hz–10 kHz). Data were acquired using a PCI-6071E, National Instruments card at 25 kHz sampling rate and were recorded together with electromyographic activity (5kHz), eye movement signals, and times of all task events and the home pad, object displacement and sensor signals (1kHz). PTNs were recorded in the right hemisphere of M43, and left hemisphere of M47.
EMG: Recordings were made from the muscles listed in the legend to Fig. 1. Data were bandpass filtered between 30 and 500 Hz (4th order Butterworth), rectified, averaged over trials and then smoothed using a 100 ms moving window.

ICMS: an isolated stimulator (Neurolog NL800 stimulus isolator, Digitimer, UK) was used to deliver trains of 13 pulses at 333 Hz, intensity typically up to 50-60 µA, duty cycle 0.5 Hz.

PT stimulation: the search stimulus was 250-300 µA (biphasic pulse, each phase 0.2 ms).

Spike Discrimination

To detect spikes we used simple thresholding applied to software filtered data (acausal 4th order, elliptic, 300Hz-3kHz). Single neurons were clustered using modified *Wave_clus* software [4]. We used an extended set of features which included not only wavelet coefficients but also the first three principal components. Spike shapes of PTNs obtained after clustering were checked against shapes of spontaneous spikes which collided antidromic spikes during PT stimulation. This was confirmed both before and after recording activity during task performance [1]. During spike discrimination, a very short (200 µs) 'dead' time between two consecutive spike events was used which allowed detection of different units which fired close together in time. For bursting units, clusters with minimum 1 ms interspike interval were accepted; for other units a minimum interspike interval of 2 ms was set.

Spike-Triggered Averaging of EMG

For M47, averages were made for each PTN from all discriminated PTN spikes and EMG recorded during the task. The identification of CM cells used the criteria employed in earlier studies from this laboratory

[5]. EMG from each muscle recorded simultaneously with the PTN, was full-wave rectified and averaged with respect to spike discharge over a period -20 ms before and 40 ms after spike discharge. Averages were compiled with a minimum of 2000 spikes. This procedure was not carried out in M43 because too few spikes were available for compiling averages.

Statistical Analysis of PTN Firing Rates

For M47, to test whether a cell showed any modulation of firing rate during action observation, we used a one-way ANOVA for three phases of the task: baseline (500 ms before the GO cue), reach (HPR to DO) and hold (H_{ON} to H_{OFF}). We performed a Bonferonni corrected posthoc test in order to compare the neuronal activity relating to the experimenter's movements (reach and hold) with the static presentation of the experimenter's object (baseline).

For M43, we compared modulation of firing rate during the 500 ms before the onset of the experimenter's movement (HPR) with the 1000 ms period centred on the time of grasp (sensor signal). For execution, we confirmed that PTNs modulated their firing rate during the monkey's grasp.

For graphical display in Fig. 3, we smoothed the average time course of each PTN's discharge and normalised it by subtracting baseline activity, and then dividing by its absolute maximum, defined using execution and observation trials.

We correlated the average firing rate during movement (between the beginning of movement (HPR) and the object displacement onset (DO)) with the movement time, which is equivalent to the speed of movement given constant distance from home pad to the object. We estimated this correlation for each cell separately for observation and execution trials, and for both sets of trials. We found 5 cells that showed significant correlation ($p<0.05$) between firing rate and movement time, 3 for execution trials and 2 for observation trials. But neither of these cells showed slope of the same sign across observation trials, execution trials and both sets of trials. Therefore the difference in firing rate between execution and observation trials could not be explained purely by the modulation of cell discharge with movement speed.

Eye Movements

In M47 we recorded the eye movements with a non-invasive ISCAN camera system (ETL-200, 120Hz). We found no correlation between the pattern of PTN activity and eye movements, although a few PTNs show a significant correlation between the time the monkey spent looking at the object and the neuronal firing rate. The monkey spent less time looking at the object during observation than during execution. However, the object fixation pattern between both conditions was highly correlated (0.92, $p<0.05$) emphasising that the monkey paid attention to the experimenter's actions during observation trials although this was not explicitly required in the task design.

Histology

At the end of the experiment in M43, the monkey was killed by an overdose of pentobarbitone (50 mg kg^{-1} i.p. Euthanal; Rhone Merieux) and perfused through the heart. The cortex and brain stem were photographed and removed for histological analysis. The implanted electrode tips were confirmed to be in the PT. M47 is still alive.

Supplemental References

1. Kraskov, A., Dancause, N., Quallo, M.M., Shepherd, S., and Lemon, R.N. (2009). Corticospinal neurons in macaque ventral premotor cortex with mirror properties: a potential mechanism for action suppression? Neuron *64*, 922-930.
2. Brochier, T., Spinks, R.L., Umilta, M.A., and Lemon, R.N. (2004). Patterns of muscle activity underlying object-specific grasp by the Macaque monkey. Journal of Neurophysiology *92*, 1770-1782.
3. Olivier, E., Baker, S.N., Nakajima, K., Brochier, T., and Lemon, R.N. (2001). Investigation into non-monosynaptic corticospinal excitation of macaque upper limb single motor units. J Neurophysiol *86*, 1573-1586.
4. Quian Quiroga, R., Nadasdy, Z., and Ben-Shaul, Y. (2004). Unsupervised spike detection and sorting with wavelets and superparamagnetic clustering. Neural. Comput . *16*, 1661-1687.
5. Quallo, M.M., Kraskov, A., and Lemon, R.N. (2012). The activity of M1 corticospinal neurons during tool use by macaque monkeys. J. Neurosci. *32*, 17235-17364.
